# Evaluation of serum and urine biomarkers for severe COVID-19

**DOI:** 10.3389/fmed.2024.1357659

**Published:** 2024-03-06

**Authors:** Yaroslav D. Shansky, Oleg O. Yanushevich, Alina V. Gospodarik, Igor V. Maev, Natella I. Krikheli, Oleg V. Levchenko, Andrew V. Zaborovsky, Vladimir V. Evdokimov, Alexander A. Solodov, Petr A. Bely, Dmitry N. Andreev, Anna N. Serkina, Sulejman S. Esiev, Anastacia V. Komarova, Philip S. Sokolov, Aleksei K. Fomenko, Mikhail K. Devkota, Sergei V. Tsaregorodtsev, Julia A. Bespyatykh

**Affiliations:** ^1^Laboratory of Molecular Medicine, Center of Molecular Medicine and Diagnostics, Federal Research and Clinical Center of Physical-Chemical Medicine of Federal Medical Biological Agency, Moscow, Russia; ^2^Federal State Budgetary Educational Institution of Higher Education "Russian University of Medicine" of the Ministry of Health of the Russian Federation, Moscow, Russia; ^3^Department of Expertise in Doping and Drug Control, Mendeleev University of Chemical Technology of Russia, Moscow, Russia

**Keywords:** COVID-19, serological tests, cubilin, XPNPEP2 protein, ORP150 protein, apolipoprotein A-I, heparin cofactor II, CREB3L3 protein

## Abstract

**Introduction:**

The new coronavirus disease, COVID-19, poses complex challenges exacerbated by several factors, with respiratory tissue lesions being notably significant among them. Consequently, there is a pressing need to identify informative biological markers that can indicate the severity of the disease. Several studies have highlighted the involvement of proteins such as APOA1, XPNPEP2, ORP150, CUBN, HCII, and CREB3L3 in these respiratory tissue lesions. However, there is a lack of information regarding antibodies to these proteins in the human body, which could potentially serve as valuable diagnostic markers for COVID-19. Simultaneously, it is relevant to select biological fluids that can be obtained without invasive procedures. Urine is one such fluid, but its effect on clinical laboratory analysis is not yet fully understood due to lack of study on its composition.

**Methods:**

Methods used in this study are as follows: total serum protein analysis; ELISA on moderate and severe COVID-19 patients’ serum and urine; bioinformatic methods: ROC analysis, PCA, SVM.

**Results and discussion:**

The levels of antiAPOA1, antiXPNPEP2, antiORP150, antiCUBN, antiHCII, and antiCREB3L3 exhibit gradual fluctuations ranging from moderate to severe in both the serum and urine of COVID-19 patients. However, the diagnostic value of individual anti-protein antibodies is low, in both blood serum and urine. On the contrary, joint detection of these antibodies in patients’ serum significantly increases the diagnostic value as demonstrated by the results of principal component analysis (PCA) and support vector machine (SVM). The non-linear regression model achieved an accuracy of 0.833. Furthermore, PCA aided in identifying serum protein markers that have the greatest impact on patient group discrimination. The study revealed that serum serves as a superior analyte for describing protein quantification due to its consistent composition and lack of organic salts and drug residues, which can otherwise affect protein stability.

## 1 Introduction

The new coronavirus disease, COVID-19, is an acute infection that mainly causes respiratory system lesions. The utility of serologic testing for severe acute respiratory syndrome coronavirus 2 (SARS-CoV-2) has been extensively debated among clinicians and laboratorians (1). Currently, its role in clinical settings remains fairly limited, with little change observed since the onset of the pandemic. The targeted molecules are those associated with the severity and mortality of COVID-19 in patients (2, 3).

The COVID-19 infection, caused by the SARS-CoV-2 virus, has a systemic impact on human organisms. It affects not only the respiratory system but also the cardiovascular, nervous, and immune systems in its pathogenesis. A number of protein molecules, such as enzymes, cofactors, and signaling molecules, play roles in the cascade of biochemical reactions triggered by the pathogen. Many of these molecules are specific to certain processes such as inflammation and hemostasis. This specificity allows their levels in human biological fluids to be targeted as potential markers of COVID-19 severity.

Multiplexed approaches, which involve the assessment of a combination of several proteins, are a focal point of interest in clinical medicine. They serve as an alternative to the traditional measurement of a single unique marker in both normal and pathological conditions. This approach significantly enhances the sensitivity and specificity of the analysis, and it has been utilized in a number of studies (4–7).

An important consideration in selecting candidates for multiplexed analysis is the non-invasive preparation of biological samples. In certain cases, it is preferable to use biological samples such as urine or saliva due to their atraumatic and simple collection in patients. In particular, the urinary proteome in COVID-19 is the focus of some studies (8).

The following proteins have sparked interest in COVID-19 patients: XPNPEP2, HCII, ORP150, CREB3L3, APOA1, and CUBN. They play a significant physiological role in the mechanisms of tissue lesions caused by the action of SARS-CoV-2.

Aminopeptidase P2 (XPNPEP2) is a membrane receptor with an enzymatic function (metalloprotease) that plays a role in the development of the inflammatory response. Being structurally and functionally similar to the ACE2 receptor, it is involved in a cascade of pathophysiological reactions during coronavirus infection (9). Studies have demonstrated elevated expression levels of XPNPEP2 in the lungs of COVID-19 patients compared to healthy individuals, which is consistent with the actively researched concept of a “cytokine storm” (4, 10).

Heparin cofactor II (SERPIND1, HCII) is a serine protease and serves as both a blood coagulation factor and a cofactor for heparin and dermatan sulfate. Through fibrinolytic effect, it prevents blood clotting (11). The production of HCII is regulated by interleukin-6 (IL-6) via positive feedback regulation. In the event of a “cytokine storm,” characterized by elevated IL-6 levels, the impact of the serine protease in inhibiting thrombin destruction intensifies, thereby exacerbating thrombosis (12–14).

Hypoxia-inducible protein 1 (HYOU1, ORP150) is involved in stress-mediated reactions within the endoplasmic reticulum, conferring a cytoprotective effect. This protein is abundantly produced in liver tissues. In particular, its expression has been demonstrated to increase by more than threefold during acute COVID-19 (8, 15).

Apolipoprotein A1 (APOA1) is a plasma apolipoprotein that serves as the primary carrier protein for high-density lipoproteins, commonly referred to as “good” cholesterol. According to the literature, APOA1 is considered one of the key proteins involved in the pathophysiology of COVID-19 serving as an important diagnosis and therapeutic marker for this infection (2, 16). In particular, it has previously been demonstrated that there is an inverse correlation between the severity of COVID-19 and the levels of APOA1 (17, 18).

Cubilin (CUBN) is a receptor protein for intrinsic vitamin B12 complexes. It is a functional link in the pathophysiology of chronic kidney disease, which can develop as a complication of COVID-19. The production of the pathological variant of CUBN results in malabsorption of vitamin B12 and proteinuria, particularly observed in children who have experienced a coronavirus infection (19).

A direct relationship between ORP150 and the clinical characteristics of severe COVID-19 is well-established. Thus, the level of ORP150 in the urine of severe COVID-19 patients was found to be more than three times higher than that in moderate COVID-19 patients (8, 20).

COVID-19 patients frequently exhibit albuminuria, a condition used as an indicator for the progression of renal failure and strongly linked to severe COVID-19 (21). Furthermore, the concentration of CUBN, an important regulator in tubular reabsorption, was found to be decreased in the urine of patients with severe COVID-19, indicating a potential dysregulation of reabsorption (22). Additionally, the levels of CUBN-transported ligand proteins, namely, selenoprotein P, plasminogen activator, urokinase, epidermal growth factor, galactosidase alfa, and apolipoprotein-H, were also observed to decrease in urine (23).

The relationship between antibodies to ORP150 and CUBN and the severity of COVID-19 remains unclear in the available literature. A similar lack of clarity exists regarding antibodies to HCII, CREB3L3, and XPNPEP2. However, it is worth noting that anti-HCII antibodies are utilized for diagnostic purposes to detect the cofactor in serum (24–27). Additionally, antibodies to CREB3L3 and XPNPEP2 are used to quantify their respective proteins in various biological samples such as serum, plasma, tissue homogenates, biological fluids, and cell cultures (28–32).

This study aimed to utilize non-invasive sampling of biomaterial or readily available biological fluids to assess the severity of COVID-19. It seems more logical to evaluate the antibodies targeting proteins formed in the human body. Despite the availability of commercial enzyme-linked immunoassay (ELISA) kits, there is limited literature on detecting antibodies to COVID-19-associated proteins. In particular, it has been established that immunoglobulins G to APOA1 serve as independent biomarkers for cardiovascular diseases and mortality, displaying proinflammatory and proatherogenic functions both *in vivo* and *in vitro* (33, 34). The significant presence of autoantibodies against native and lipid-free apoA1 was demonstrated after two consecutive injections of anti-SARS-CoV-2 mRNA vaccines (35). A study on the general population showed that exposure to SARS-CoV-2 elevated baseline levels of anti-apoA-1 IgG (33). These autoantibodies act as active mediators of sterile inflammation and independent predictors of general morbidity and adverse events in numerous clinical situations, generating interest in their assessment in the context of COVID-19 (8).

This study aimed to quantify antiHCII, antiAPOA1, antiCUBN, antiXPNPEP2, anti-ORP150, and antiCREB3L3 levels in both urine and serum samples from moderate and severe COVID-19 patients, followed by multiple binomial regression modeling and estimation of the diagnostic value of the multiplex model.

## 2 Materials and methods

### 2.1 Inclusion criteria and sampling

Patients hospitalized at the Russian University of Medicine and diagnosed with the “new coronavirus infection” were enrolled in this study. The median age of the patients was 56.7 years old (median 57, min–max 4–65 years). During hospitalization, the symptoms of interest included hyperthermia, thoracic pain, and respiratory distress. COVID-19 in patients was diagnosed through PCR analysis of pharyngeal and nasal swabs sampled between May and December 2021. The diagnosis was further confirmed through computed tomography scan and X-ray assays.

Drug administration in patients was tailored to correspond with the variant of COVID-19 and its severity. Initially, patients received anticoagulant medication (heparin sodium), antibiotics (penicillin, cephalosporins, macrolides, and fluoroquinolones), glucocorticoids, and vitamins (ascorbic acid). In the event of worsening general conditions, such as respiratory or heart failure, patients were transferred to the intensive care unit and provided with mechanical ventilation. The total duration of hospitalization ranged from 3 to 30 days, until discharge or resolution of clinical outcomes.

When hospitalized, all patients or their legal representatives provided informed consent for serum and urine sampling for scientific study. The study was approved by the Independent Interdisciplinary Committee on Ethical Expertise of Clinical Trials, protocol 01–21 on 28 January 2021. The patients were divided into two subgroups: (a) those with moderate COVID-19 (*n* = 20) and (b) those with severe COVID-19 (*n* = 20), based on their discharge status or placement in the intensive care unit. Urine and serum samples were collected from all patients at two different time points: (1) upon hospitalization and (2) at the onset of therapy (severe illness) or upon hospital discharge (moderate illness). A total of 40 urine samples and 30 serum samples were collected from each subgroup, resulting in 80 urine samples and 60 serum samples.

### 2.2 Preparation of the urine samples

The urine samples were initially concentrated because the concentrations of the antibodies under study were expected to be lower in this analyte than those in the serum. Concentration was achieved through three methods: centrifugation using Amicon® centrifuge filters (M1), reprecipitation after treatment with isopropyl alcohol (M2), and lyophilization (M3).

Amicon® Ultra-0.5 10 K filters were placed in the supplied 2-mL plastic tubes and loaded with 0.5 mL of urine. The tubes containing filters were then closed and centrifuged at 14,000 *g* for 10 min. Subsequently, the inserts were inverted and transferred to 2-mL plastic tubes to collect the concentrate, which was centrifuged again at 1,000 *g* for 2 min. This process yielded approximately 20 μL of concentrate from 0.5 mL of urine. The procedure was repeated until a concentrated volume sufficient for ELISA was obtained (~200 μL).

For the M2 method, the following protocol was used. Approximately 3 mL of urine was combined with 9 mL of acetone cooled to −20°C, inside a plastic centrifuge tube placed on ice. After thorough mixing, the tubes were stored at −20°C for 4 h and then centrifuged at 2,000 *g* for 10 min using an Eppendorf 5810R centrifuge (Germany). The resulting precipitate was washed with 1 mL of acetone and air-dried in an open tube. Subsequently, the residue was dissolved in 0.3 mL of phosphate buffer solution (pH 7.4) and transferred to plastic Eppendorf tubes for storage. This procedure was expected to increase the protein concentration rate by up to 10-fold.

The process of lyophilization (M3) was followed by reconstitution in phosphate buffer, which was carried out according to the following protocol. Initially, approximately 10 mL of initial urine samples was transferred into glass “penicillin” vials, which were then cooled to −20°C and placed in a Freeze Dryer 10-N (Scientz, China). The device operation consisted of two stages: (1) main drying (temperature − 70°C; vacuum depth 0.1 mbar; duration 3 h) and (2) final drying (temperature − 70°C; vacuum depth 0.001 mbar; duration 1 h). Afterward, the lyophilized residue was dissolved in 0.5 mL of phosphate buffer solution (pH 7.4) and transferred to plastic Eppendorf tubes for storage. This process was anticipated to result in a 10-fold increase in protein concentration.

The level of concentration was verified by assessing the total protein content in urine before and after the concentration process. This assessment was performed using the FURUNO CA-800 automatic biochemical analyzer (Furuno Electric Co., Ltd., Japan) using a reagent kit (F 10210 9,910 930) specifically designed for determining total protein in urine, allowing for up to 680 tests. Urine samples, both pre- and post-concentration, were aliquoted to approximately 0.2 mL and transferred into glass cuvettes for analysis, which was then placed into the analyzer.

### 2.3 Enzyme-linked immunoassay (ELISA)

The multiplexed approach was assessed through the measurement of protein content in urine. The determination method involved quantitative competitive enzyme immunoassay on a sorbent using commercial kits as follows: the AEA284Hu ELISA Kit for Anti-Heparin Cofactor II antibody (antiHCII), the AEC537Hu ELISA Kit for Anti-Hypoxia Up Regulated 1 antibody, the AEA519Hu ELISA Kit for Anti-Apolipoprotein A1 antibody, the AEC411Hu ELISA Kit for Anti-Cubilin antibody (CloudClone, China), and CREB3L3 (BlueGene Biotech, China). Sample preparation and analysis were conducted following the manufacturer’s instructions provided with the kits, using an automatic Lazurite immunoassay analyzer (Dynex, United States).

The general procedure for ELISA to detect antiHCII, antiAPOA1, antiCUBN, and antiORP150 was as follows: In a 96-well plate, appropriate wells were filled with antigens (HCII, APOA1, CUBN, XPNPEP2, HYOU1, and CREB3L3) adsorbed on the surface; 100 μL of diluted standards or samples containing the target antibodies (antiHCII, antiAPOA1, antiCUBN, and antiORP150) were added to each well and incubated at 37°C for 1 h. After incubation, the liquid was removed and 100 μL of the detection reagent was added to each well, followed by another incubation at 37°C for 1 h. Subsequently, the liquid was discarded and the wells were washed 5 times with 350 μL of wash buffer per well. After drying, 90 μL of tetramethylbenzidine was added to each well and incubated at 37°C for 15 min. Finally, 50 μL of stop solution was added to halt the reaction, and the optical density of the resulting solution was measured using a built-in photometer at a wavelength of 450 nm.

ELISA was conducted to assess the presence of antiXPNPEP2 and antiCREB3L3 according to the following protocol. In a 96-well plate, corresponding wells with an antigen adsorbed on the surface (XPNPEP2 and CREB3L3) were prepared. 100 μL of blank (phosphate buffer solution, pH 7.0–7.2), diluted standards, or samples containing target antibodies (antiXPNPEP2 and antiCREB3L3) were added to these wells. Subsequently, 50 μL of the conjugate solution was added to the wells containing samples and incubated at 37°C for 1 h. Next, the liquid was then discarded and the wells were washed 5 times with 400 μL of wash buffer per well. Next, 50 μL each of substrate solution A and substrate solution B were added to all wells, followed by incubation at 37°C for 20 min. Finally, 50 μL of stop solution was added to halt the reaction, and the optical density of the resulting solution was measured using a built-in photometer at a wavelength of 450 nm.

The concentrations of antiHCII, antiAPOA1, antiCUBN, antiXPNPEP2, antiORP150, and antiCREB3L3 in biological fluids were determined using a calibration curve built into the analyzer software.

By analyzing the concentrations of the targeted antibodies in biomaterial samples at the study's commencement of the study and conclusion, we also calculated their relative changes throughout the progression of the disease or its ongoing moderate course.

### 2.4 Statistical analysis

The data were statistically processed using the RStudio 2023.03.1 software (Posit Software, PBC). Mean values were compared between groups using the non-parametric Kruskal–Wallis test with post-hoc comparisons. Differences were considered significant at *p* < 0.05. The graphs were plotted using *ggplot2*, *ggpubr*, *ggsci*, and *ggsignif* packages. The ROC analysis was performed using *caret*, *cutpointr*, and *pROC* packages for *R*. The PCA was performed using *factoextra* and *FactoMineR* packages for *R*. The linear binomial regression model with SVM was built using *glm2* and *caret* packages for *R*.

## 3 Results

### 3.1 Clinical data

Patients included in the study were categorized into groups based on the severity of their disease course as outlined in the section Materials and Methods criteria. Lethal outcomes among severe patients were associated with escalating respiratory failure and the onset of multiorgan failure. There were two fatal outcomes in the severe patient group. Patients with moderate disease severity had an average bed stay duration of 12–14 days, while those with severe disease course stayed for more than 14 days. Throughout inpatient treatment, blood oxygenation was regularly monitored to gauge the severity of hypoxemia, and blood coagulation ability was assessed to ensure the proper selection of anticoagulants.

The total protein concentration in urine did not differ significantly among patients (*p* = 0.484), with a median of 54.21 (range 0.111–482.00) for moderate and 28.12 (range 0.111–422.00) for severe patients. However, urine samples from both groups exhibited residues of oxalate crystals, desquamated epithelium, and blood. The presence of oxalate crystals was attributed to the prolonged administration of high doses of vitamins (ascorbic acid and cholecalciferol) to patients. Meanwhile, the desquamated epithelium and blood in the urine of severe cases were likely a result of analyte sampling using transurethral catheters. These impurities posed a challenge to the accurate quantification of total protein content in the urine. The reduced solubility of the proteins in the presence of organic anions led to incorrect normalization of the content of studied antibodies to total protein.

### 3.2 Urine concentration rate

The use of Amicon® centrifuge filters allows concentration of the proteins within 1–3 orders of magnitude (median concentration factor 34.75, range 1.5–46055.0), without exposing them to chemicals that may be incompatible with the components of the ELISA kits or cause protein denaturation. The variation in concentration multiplicity is associated with different qualitative and quantitative protein compositions of the raw urine.

The precipitation method with acetone followed by redissolution results in a less effective protein concentration in urine (multiplicity median 4.85; mix–max 1.2–4.8). The lower yield of the concentrated protein is attributed to the denaturation of many proteins in the presence of acetone.

The lyophilization method achieves a more effective protein concentration compared to acetone precipitation (median fold 17.4; range 33.5–368.0). However, the lyophilization method is also not universally gentle on all proteins; some may undergo denaturation, affecting their final yield and solubility.

**Figure 1:**
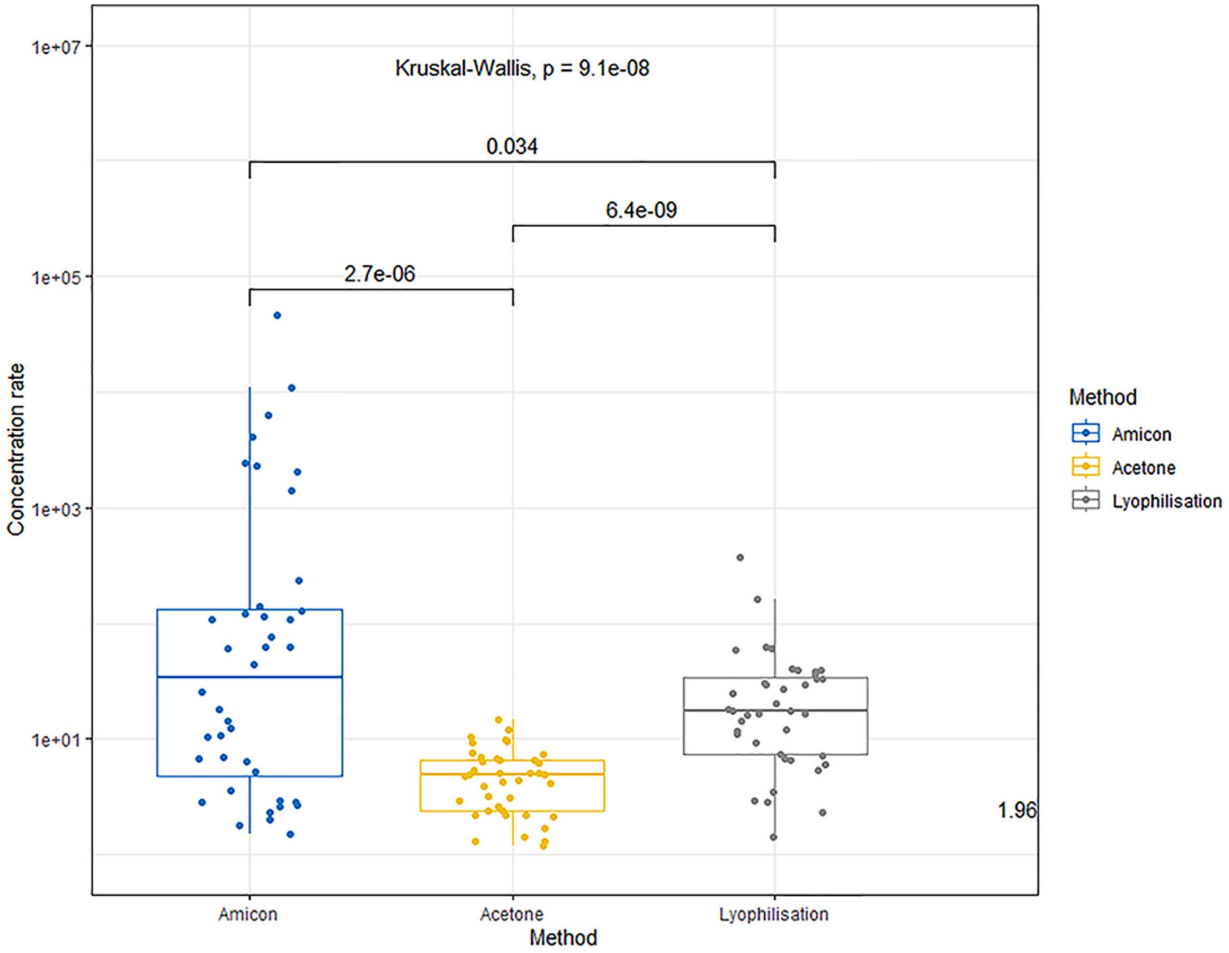
Comparative concentration fold of urine samples using various concentration methods.

The comparison of urine concentration levels using the aforementioned methods is illustrated in Figure 1.

### 3.3 ELISA results

**Figure 2:**
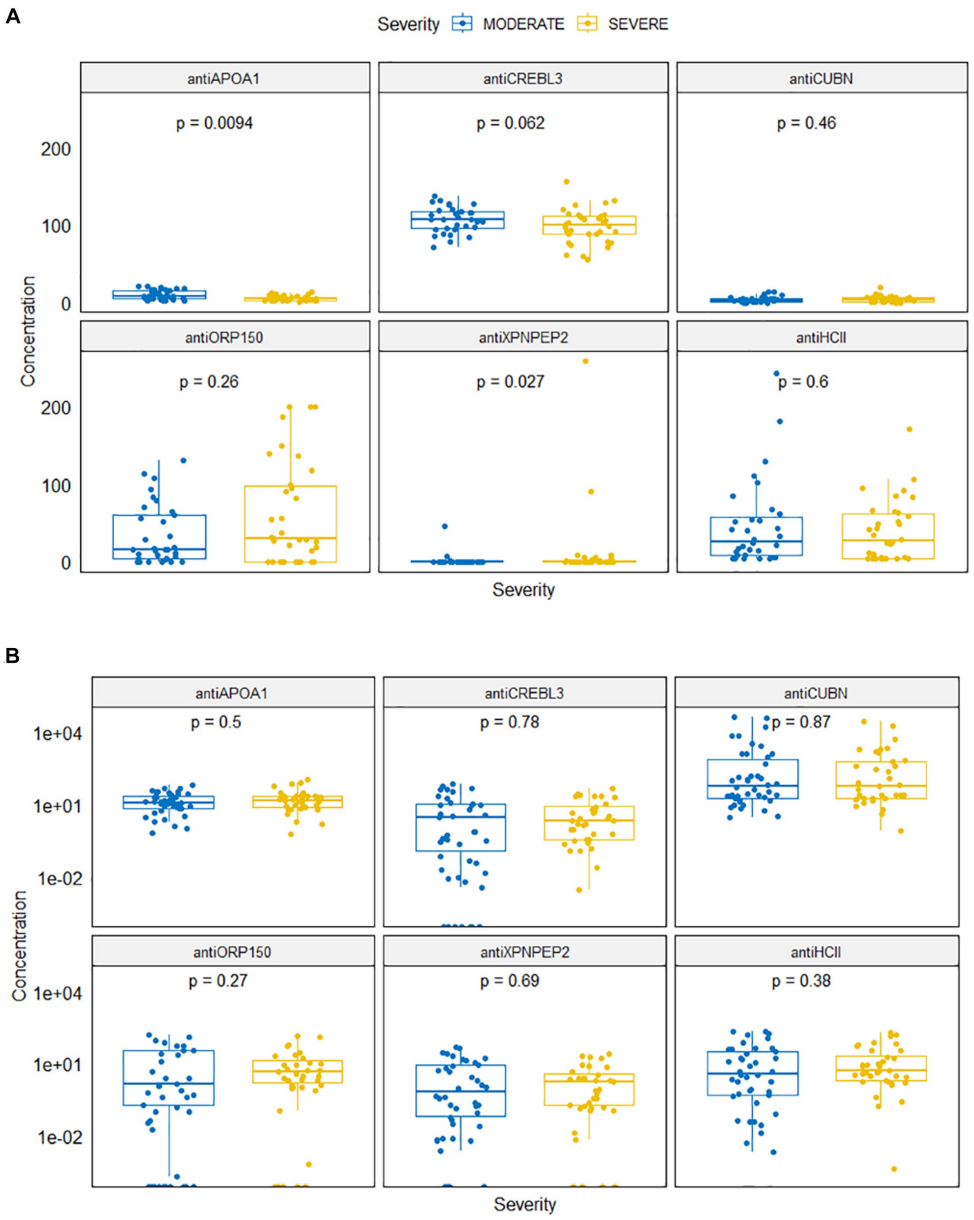
Antibody levels in serum **(A)** and in urine **(B)** samples for moderate and severe COVID-19 patients.

The concentrations of antiHCII, antiAPOA1, antiCUBN, antiXPNPEP2, antiORP150, and antiCREB3L3 are shown in Figure 2. Slightly elevated levels of biomarkers were observed in urine compared to serum for antiCUBN, antiXPNPEP2, and antiAPOA1. Conversely, antiHCII, antiORP150, and antiCREB3L3 exhibited the opposite trend.

Levels of antiAPOA1 and antiXPNPEP2 were different for patients with severe and moderate COVID-19 (Figure 2A). The mean antiAPOA1 level was 5.47 ng/mL (median 4.82 ng/mL, min–max 1.98–21.12 ng/mL) in severe patients, compared to 9.47 ng/mL (8.03 ng/mL, 1.15–13.93 ng/mL) in moderate patients; this difference was statistically significant (*p* = 0.0094). The antiXPNPEP2 level was higher in severe COVID-19 patients: 11.268 (0.0–257.94) compared to 1.63 (0.00, 0.00–44.96) in moderate COVID-19 patients, with a *value of p* of 0.027.

The serum levels of antiHCII, antiCUBN, antiORP150, and antiCREBL3L3 did not differ significantly in both groups (*p* = 0.600, *p* = 0.46, *p* = 0.26, and *p* = 0.062, respectively). The mean concentration of antiHCII was 46.44 ng/mL (median 25.33; range 3.43–242.82) in moderate COVID-19 patients and 38.55 ng/mL (26.99, 3.43–170.52) in severe COVID-19 patients. The concentration of antiORP150 varied slightly between severe 60.55 ng/mL (29.49, 0.00–199.73) and moderate 34.51 ng/mL (15.33, 0.00–130.25) patients. However, the concentration of antiCREBL3 was consistently high in both moderate 107.14 ng/mL (107.79, 71.15–137.69) and severe 98.23 ng/mL (100.05, 54.79–155.54) patients. Conversely, the level of antiCUBN was low in both groups: 3.87 ng/mL (2.61, 0.00–13.34) in moderate patients and 4.50 ng/mL (3.74, 0.00–19.69) in severe patients.

Regarding urine samples, there was no statistically significant difference in protein levels between groups (Figure 2B). The concentrations of antiHCII, antiORP150, and antiAPOA1 in urine were 31.00 ng/mL (4.01, 0.00–216.44), 17.17 ng/mL (0.39, 0.00–153.64), and 17.43 ng/mL (12.73, 0.67–64.72) in moderate COVID-19 patients, respectively, and those were 26.45 ng/mL (5.48, 0.00–186.83), 15.29 ng/mL (3.12, 0.00–136.43), and 21.65 ng/mL (15.93, 0.63–113.36) in severe COVID-19 patients, respectively. Similarly, the concentrations of antiCREBL3L3 and antiXPNPEP2 were 10.29 ng/mL (0.46, 0.00–77.89) vs. 6.46 ng/mL (0.39, 0.00–47.32) and 7.91 ng/mL (2.19, 0.00–49.72) vs. 3.82 ng/mL (0.83, 0.00–25.21) for both groups, respectively. However, a number of abnormally high values were observed for antiCUBN concentrations: 3067.74 ng/mL (60.34, 3.18–43701.97) in moderate COVID-19 patients compared to 1877.33 ng/mL (61.27, 0.87–29859.44) in severe COVID-19 patients.

**Figure 3:**
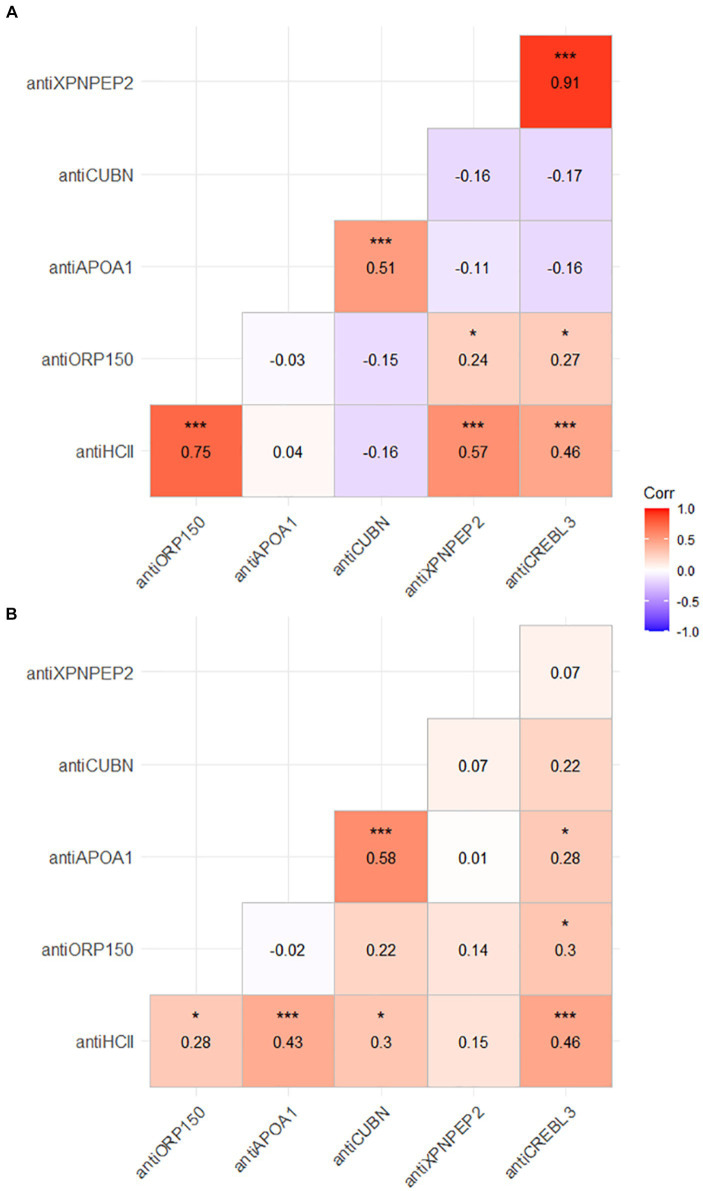
Correlation of protein markers in urine **(А)** and serum of patients **(В)**.

Several proteins correlated with each other in both urine and serum samples. The strongest and most statistically significant correlation was observed for antiXPNPEP2 and antiCREBL3 urine levels (*r* = 0.91, *p* < 0.001). In urine samples, significant correlations were observed for antiHCII–antiXPNPEP2 (*r* = 0.75, *p* < 0.001), antiHCII–antiXPNPEP2 (*r* = 0.57, *p* < 0.001), antiHCII–antiCREBL3 (*r* = 0.46, *p* < 0.001), and antiAPOA1–antiCREBL3 (*r* = 0.51, *p* < 0.001) pairs (Figure 3A). In blood serum, fewer correlations were observed among the protein markers, yet they were statistically significant as well (Figure 3B). The antiHCII level significantly correlated to antiORP150 (*p* < 0.05), antiAPOA1 (*p* < 0.001), antiCUBN (*p* < 0.05), and antiCREBL3 (*p* < 0.001), though these correlations were relatively weak (*r* = 0.28, 0.43, 0.3, and 0.46, respectively). The only antiXPNPEP2–antiHCII correlation was weak and non-significant (*r* = 0.15, *p* > 0.05), both the correlations of antiXPNPEP2 to other markers in serum. In addition, antiCREBL3 correlated significantly to antiOR150 (*r* = 0.3, *p* < 0.05) and antiAPOA1 (*r* = 0.28, *p* < 0.05) as well as antiAPOA1 to antiCUBN (*r* = 0.58, *p* < 0.001); the last one is the strongest correlation for patients’ serum as an analyte.

### 3.4 Principal components analysis (PCA)

Taking these strong correlations into account (see 3.3 section), further PCA was performed to reduce the dimensions and describe the difference between experimental groups.

**Figure 4:**
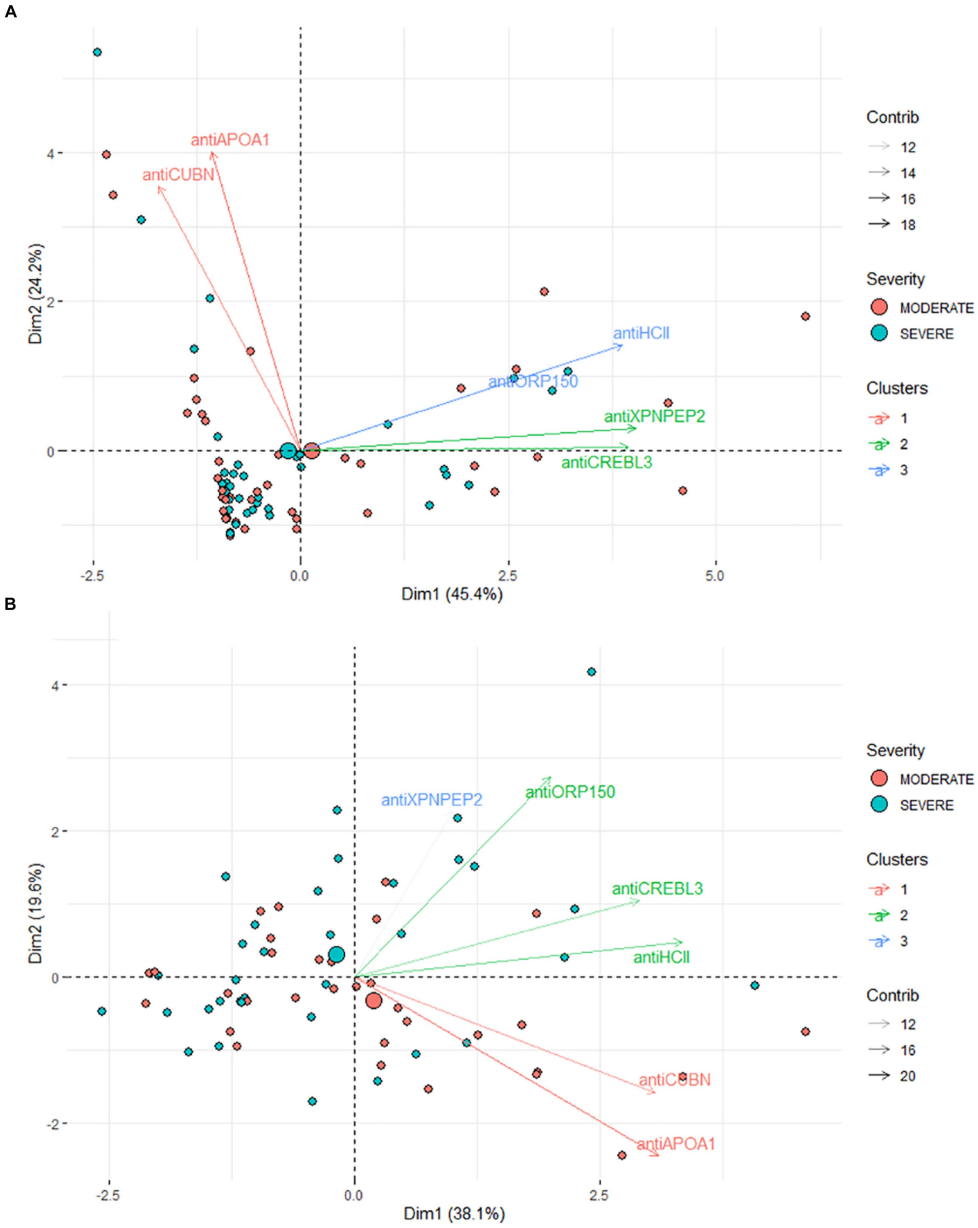
**(A)** Results of PCA for urine samples. **(B)** Results of PCA for blood serum samples. The markers are clustered according to their coordinates in PC1–PC2 dimensions.

PCA revealed no clear distinction between patient groups when assessing protein levels in urine (Figure 4A). Both moderate and severe patients appear as a single large cluster in the two-dimensional plot. In particular, antiCREBL3 and antiXPNPEP2 exhibit the highest loading onto the first principal component (PC) and are tightly correlated, forming a distinct cluster of variables. Conversely, antiHCII shows significant loading on both PC1 and PC2 and forms a second cluster of variables along with antiORP150, correlating with the last one. The antiCUBN and antiAPOA1 proteins have a main loading to PC2 and much less to PC1, forming a third cluster of variables.

In contrast, for patients’ serum, a more pronounced difference between the two groups is evident in the PC1–PC2 dimension. These components collectively account for 52.4% of the total variance (Figure 4B), making the stratification of moderate and severe COVID-19 patients challenging, albeit less so than in urine samples. The most significant loadings were observed for antiORP150, antiAPOA1, and antiCUBN proteins, with support by their area under the curve (AUC) values. The levels of antiHCII, antiORP150, and antiCUBN correlate, though not as strongly as in urine samples. AntiXPNPEP2 contributes minimally compared to other proteins. AntiCREBL3 and antiHCII contribute mostly to PC1, forming one cluster of variables, whereas antiXPNPEP2 and antiORP150 contribute to PC1 and PC2, but not equally. Interestingly, the antiAPOA1 and antiCUBN contribute both to PC1 and PC2, but the loading to PC2 is in a negative manner.

### 3.5 ROC analysis

**Figure 5:**
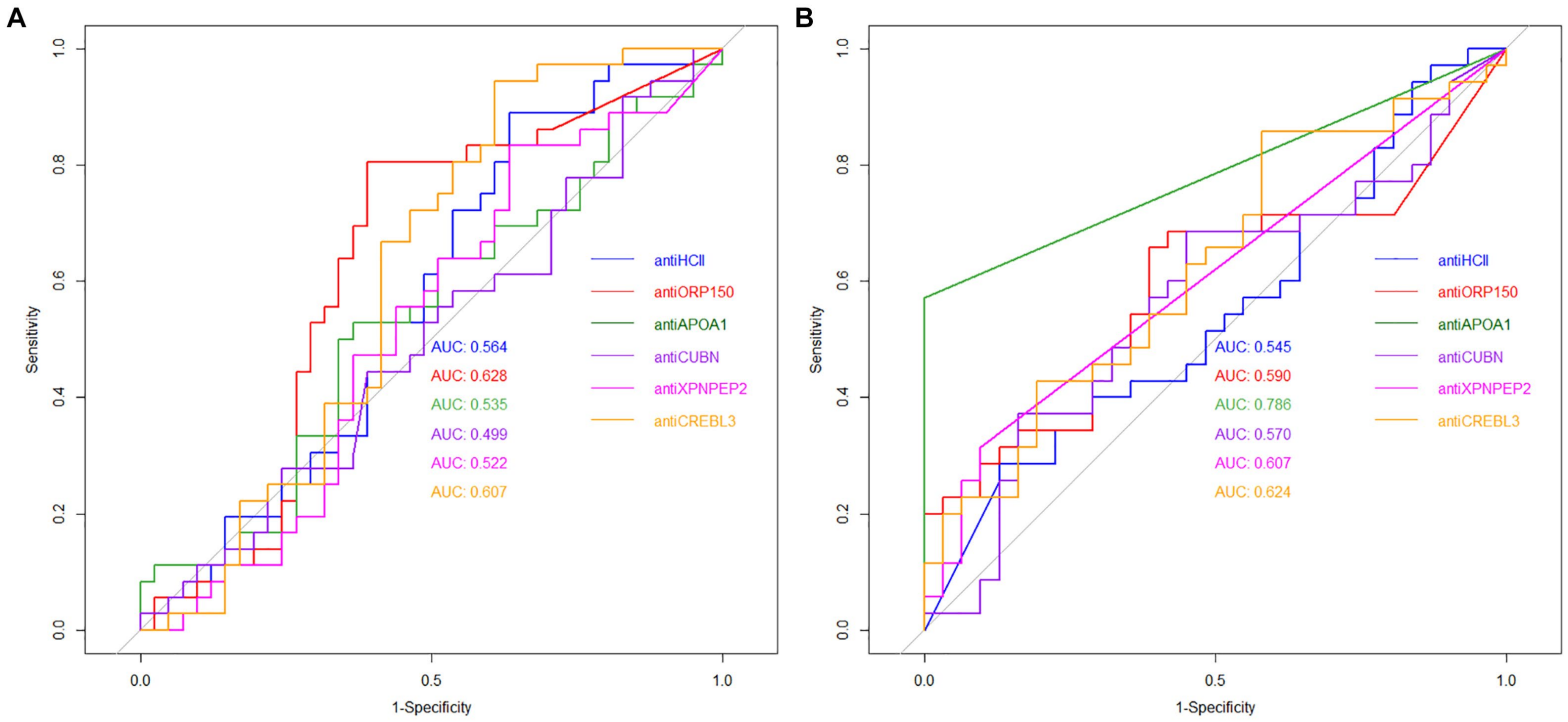
ROC curves for unique protein antibodies in urine **(A)** and blood serum **(B)** of patients.

The diagnostic value of unique proteins in blood serum and urine is relatively low, ranging from 0.499 to 0.786 (Figure 5A). When urine is used as an analyte, the highest AUC value was observed for antiORP150 (0.628) and antiCREBL3 (0.607). When measuring antiHCII, antiCUBN, antiAPOA1, and antiXPNPEP2 in urine, the AUC values were approximately 0.5. The corresponding values for sensitivity and specificity are also shown in Table 1. In particular, the classification of patients with COVID-19 based on these antibody levels in urine is deemed ineffective.

**Table 1:** Diagnostics characteristics of protein antibodies in blood serum and urine.

Antibody	Blood serum				Urine			
	AUC	Cut-off	Specificity	Sensitivity	AUC	Cut-off	Specificity	Sensitivity
*antiHCII*	0.634	>0.511	0.375	0.375	0.558	>4.638	0.476	0.528
*antiORP150*	0.583	<0.722	0.500	0.500	0.628	>1.410	0.366	0.639
*antiAPOA1*	0.517	<0.446	0.375	0.632	0.545	>13.551	0.452	0.556
*antiCUBN*	0.556	<0.933	0.3125	0.421	0.511	>61.267	0.500	0.500
*antiXPNPEP2*	0.794	>0.035	0.909	0.294	0.515	>0.518	0.452	0.556
*antiCREBL3*	0.484	<0.167	0.091	0.091	0.600	>1.500	0.429	0.583

In contrast, the highest AUC value of 0.786 was observed for antiAPOA1, when determined in blood serum, indicating a strong predictive model. For antiXPNPEP2, antiCREBL3, antiHCII, and antiCUBN, the AUC values ranged from 0.545 to 0.624, respectively (Figure 5B). Thus, only antiAPOA1 level in blood serum is informative enough to discriminate the moderate and severe COVID-19 (good predictability). The antiXPNPEP2 and antiCREBL3 levels demonstrate moderate predictability, whereas antiHCII and antiCUBN levels exhibit insufficient predictive value (Table 1).

Additionally, a binary linear regression model was employed to assess the diagnostic characteristics of protein antibody combinations. The model was created using *glm2* package for R. The following combinations of the protein markers were evaluated: antiAPOA1 + antiCUBN, antiAPOA1 + antiCUBN + antiHCII, antiAPOA1 + antiCUBN + antiНСII + antiORP150, antiAPOA1 + antiHCII + antiCUBN+ antiCREBL3 + antiORP150, and all six markers combined. The regression equation had the following form:


$$\begin{array}{l} y\left(x\right)=a+b\times \mathrm{antiORP}150+c\times \mathrm{antiCREBL}3+d\times \mathrm{antiXPNPEP}2\\ \;+e\times \mathrm{antiAPOA}1+f\times \mathrm{antiHCII}+g\times \mathrm{antiCUBN} \end{array}$$

The values of the coefficients are presented in Table 2.

**Table 2:** Parameters of multiple linear regression equations.

Biological sample	Antibody combination	Coefficients						
		a	b	c	d	e	f	g
Serum	*antiAPOA1 + antiCUBN*	1.315	–	–	–	0.439	–	0.451
	*antiAPOA1 + antiCUBN + antiHCII*	1.298	–	–	–	0.495	0.007	0.468
	*antiAPOA1 + antiCUBN + antiHCII + antiORP150*	1.041	0.007	–	–	0.464	0.004	0.443
	*antiAPOA1 + antiHCII + antiCUBN+ antiORP150 + antiCREBL3*	4.541	0.008	−0.037	-	−0.497	0.012	0.463
	*All*	8.430	0.024	−0.066	1.440	−0.921	0.021	0.714
Urine	*antiAPOA1 + antiCUBN*	−0.466	–	–	–	0.023	-	−5.5 × 10^−5^
	*antiAPOA1 + antiCUBN + antiHCII*	−0.390	–	–	–	0.025	−0.004	−6.4 × 10^−5^
	*antiAPOA1 + antiCUBN + antiHCII + antiORP150*	−0.402	0.003	–	–	0.025	−0.005	−6.4 × 10^−5^
	*antiAPOA1 + antiHCII + antiCUBN+ antiORP150 + antiCREBL3*	−0.356	−0.010	−0.005	–	0.024	−0.004	−6.4 × 10^−5^
	*All*	−0.369	−0.010	0.064	−0.147	0.024	0.008	−6.4 × 10^−5^

**Figure 6:**
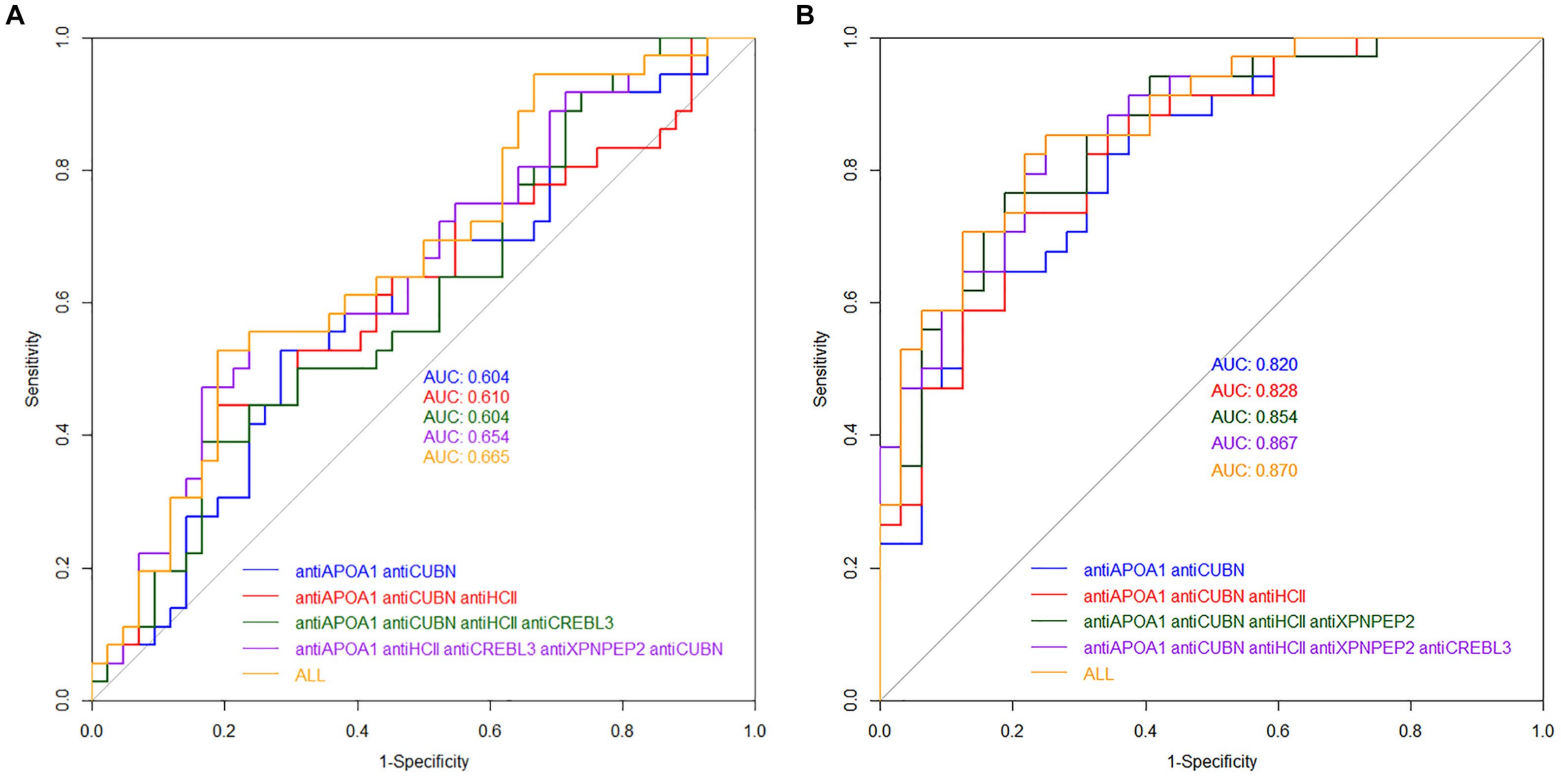
ROC curves for combined protein antibodies in urine **(A)** and blood serum **(B)** of patients.

The AUC values for models of protein combinations when determined in urine still exhibited moderate or lower diagnostic value. The highest AUC value observed was 0.658 for all six markers. The combination of antiAPOA1, antiCUBN, and antiHCII had an AUC of 0.607. The other two combinations showed below moderate AUC values (Figure 6A).

Simultaneous evaluation of multiple proteins significantly enhances the diagnostic value of the test when performed on blood serum (Figure 6B). Other combinations had AUC values higher than 0.700. In particular, the combination of antiAPOA1, antiCUBN, and antiHCII exhibited a high AUC value of 0.828, along with sensitivity and specificity values of 0.735 and 0.8125, respectively. Increasing the number of markers up to six slightly improved the diagnostic value, with a maximal AUC of 0.870, specificity of 0.781, and sensitivity of 0.823, achieving when all six proteins were significant. These findings align with PCA data, where antiHCII contributes more to the principal components than antiORP150 and antiCUBN. Moreover, the levels of antiHCII and antiCREBL3 exhibit a strong correlation, suggesting that using antiHCII alone in ROC analysis is sufficient. Cutoff values and corresponding sensitivity and specificity values for protein combinations are presented in Table 3.

**Table 3:** Prognostics characteristics of protein antibodies when combined in blood serum and urine.

Antibody combination	Blood serum				Urine			
	AUC	Cut-off	Specificity	Sensitivity	AUC	Cut-off	Specificity	Sensitivity
*antiAPOA1 + antiCUBN*	0.820	>0.617	0.875	0.647	0.604	>0.471	0.715	0.528
*antiAPOA1 + antiCUBN + antiHCII*	0.828	>0.595	0.812	0.735	0.610	>0.496	0.810	0.444
*antiAPOA1 + antiHCII + antiCUBN+ antiORP150*	0.839	>0.572	0.812	0.735	0.604	>0.494	0.810	0.444
*antiAPOA1 + antiHCII + antiCUBN+ antiCREBL3 + antiORP150*	0.858	>0.509	0.781	0.794	0.593	>0.423	0.357	0.833
*All*	0.870	>0.506	0.781	0.823	0.665	>0.515	0.809	0.528

The accuracy of the regression model was assessed using the support vector machine (SVM) learning method with a linear classifier. For blood serum samples, 80% of the dataset was used for building the predictive model (*n* = 53), while 20% was reserved for evaluating the model (*n* = 13). For urine samples, the testing set and test set sizes were *n* = 62 and *n* = 16, respectively, maintaining the aforementioned percentage split.

**Figure 7:**
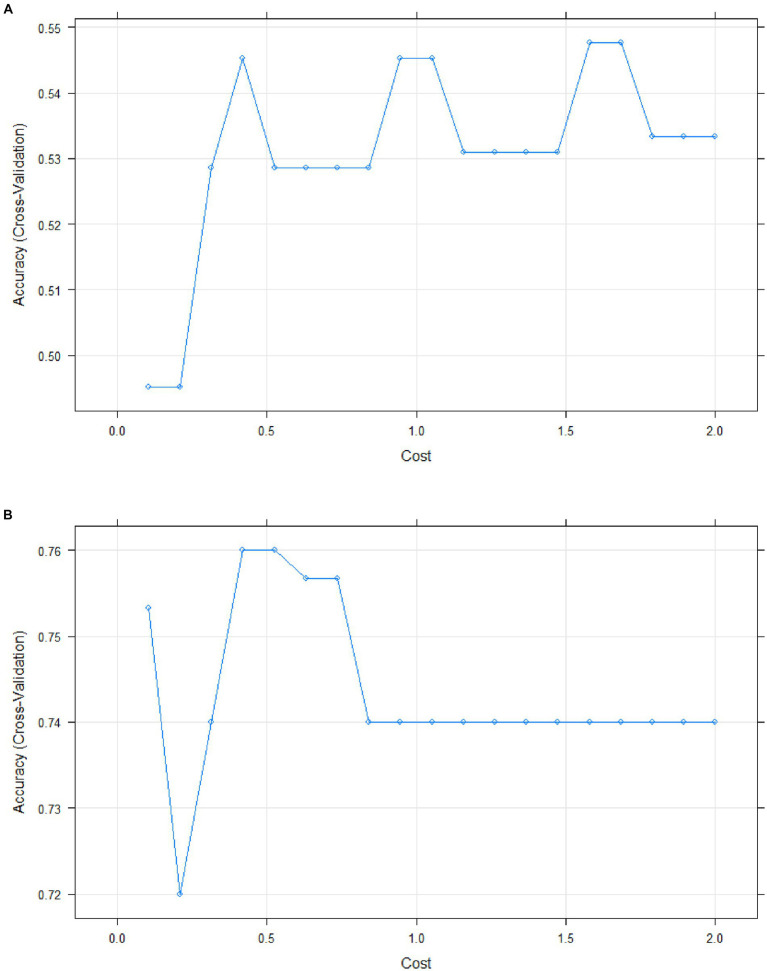
Model accuracy plot for urine **(A)** and serum **(B)**.

The accuracy rate of model reached 0.667 for urine (Figure 7A). Additionally, the quadratic discriminant analysis indicated that the accuracy of the predictive model was 0.591.

In contrast, blood serum was identified as the optimal biological material for measuring protein levels, with a model accuracy rate of 0.833 (Figure 7B). The quadratic discriminant analysis has shown the accuracy of the predictive model to be 0.833 as well.

## 4 Discussion

In the present study, we evaluated the content of antibodies, specifically protein serological markers, associated with moderate and severe variants of COVID-19. The focus of the study was on antibodies targeting molecules associated with COVID-19, an area that has been insufficiently studied, particularly in the field of clinical diagnosis. Given their multifaceted role in COVID-19 pathogenesis, we compared antibody levels for six protein molecules commonly found in blood serum. These proteins were detected using ELISA with commercially available kits. A multiple regression approach was successfully employed to discriminate the severity groups among COVID-19 patients. The analysis demonstrated that the predictive model achieved appropriate levels of accuracy, sensitivity, and specificity, making it suitable for clinical application.

The fast and precise diagnosis of COVID-19 is essential for preventing serious complications such as respiratory and cardiac failure. An ideal diagnostic test should possess optimal diagnostic properties including, specificity, sensitivity, and precision (1), while also being non-invasive and providing rapid results.

Various serological tests are available for diagnosing COVID-19, each employing different techniques such as direct or indirect enzyme-linked immunosorbent assay (ELISA) (36–38), immunochromatographic assay (39, 40), chemiluminescent immunoassay (CLIA) (38, 41–43), lateral flow analysis (44–46), and others. All these techniques aim to detect specific antibodies to the SARS-CoV-2 virus. Currently, immune-based diagnostics remain the primary approach for COVID-19 diagnosis. However, these methods are often time-consuming and cumbersome when rapid diagnosis is imperative.

Taking these issues into account, numerous solutions for “point-of-care” diagnosis of COVID-19 were developed, including test strips, among other methods. Unfortunately, their utility in clinical and laboratory settings is limited. First, they only detect a restricted number of markers, such as viral RNA. Second, they require specialized equipment to generate an appropriate analytical signal. Third, the use of novel platforms results in additional costs. Therefore, the fastest approach, which could potentially be integrated with routine clinical laboratory tests, is to identify the specific biochemical markers in blood serum. Numerous non-immunological markers have been reported in infected patients, including hematological parameters correlated to COVID-19 severity, such as blood cell counts and their ratios (e.g., neutrophil-to-lymphocyte ratio; neutrophil-to-CD8+ T-cell ratio) (47). However, these markers lack specificity overall, although they may be useful for patient follow-up. Biochemical parameters also hold diagnostic value, particularly in multiplex studies, including alanine aminotransferase, aspartate aminotransferase, γ-glutamyltransferase, urea, and creatinine (48). Nevertheless, these markers are involved in various pathogenetic pathways, besides COVID-19. Protein markers associated with COVID-19 pathogenesis appear to be attractive and informative, especially given the possibility of determining them in blood serum. However, there is a lack of data regarding the diagnostic characteristics of antibodies against these proteins. Therefore, we aimed to assess such antibodies, as well as their combinations, in moderate and severe COVID-19 patients.

Multiplexed approaches have already been employed to predict certain characteristics of COVID-19 and identify informative markers (49, 50). These approaches enable the numerical discrimination of groups with high accuracy, often utilizing multiple regression modeling. At the same time, it is essential to use markers that have already been identified as functional links to the COVID-19 pathogenesis, while also excluding incidental dependencies between characteristics.

First, we assessed the protein levels in urine. The levels of all unique protein biomarkers in the urine were found to be non-discriminative. There was no significant difference observed between moderate and severe COVID-19 patients for any of the protein markers (Figure 2B). The presence of prescribed medication and various inorganic and organic ions, such as sodium chloride, creatinine, or oxalate (attributed to the prescription of ascorbic acid and other vitamins), appears to be the reason for the change in protein levels change in urine. Consequently, calcium oxalate along with urine proteins contributes to the formation of kidney stones (51, 52). Thus, despite the non-invasive nature of urine collection, this sample was deemed insufficiently informative for diagnosing severe COVID-19. Additionally, ROC analysis confirmed, that even when multiple proteins are used, the diagnostic value of a multiple regression model is insufficient, with the AUC values hovering approximately 0.600 even for all six markers (Figure 6A).

It’s important to note that the level of urine biomarker needs to be normalized to urinary creatinine to account for the patient’s hydration status. However, creatinine adjustment in point-of-care diagnostics presents a challenge. Its small molecule size does not induce specific antibody production, such as in immunoassays (53). Moreover, its level depends on factors like body weight and diet, the latter of which introduces exogenous influences on creatinine levels (54). Meanwhile, it should be noted that COVID-19 severity could also contribute to variations in urine protein levels as proteinuria is influenced by the severity of coronavirus infection as well (12, 55).

In this study, the total protein level did not significantly differ between moderate and severe patients (*p* < 0.484), although the limited sample size should be taken into account. This limitation might further affect the appropriate normalization of targeted molecules. Therefore, the search for a parameter to normalize antibody levels in the urine of moderate and severe COVID-19 patients remains an ongoing issue. To address this, we plan to obtain missing data in follow-up studies and normalize the data on urine creatinine as well.

The majority of unique protein biomarker levels in blood serum did not differ significantly between patient groups, except for antiXPNPEP2 and antiAPOA1 (Figure 2A). The antiXPNPEP2 was upregulated (*p* = 0.037) in severe COVID-19 patients, whereas antiAPOA1 was downregulated in this group (*p* < 0.001). However, their diagnostic value separately was insufficient to distinguish between patient groups. Specifically, antiXPNPEP2 exhibited very good specificity (0.909), but low sensitivity (0.294), while the best values for these parameters were 0.375 and 0.632, respectively, for antiAPOA1.

The PCA method demonstrated that two principal components adequately described the samples, with antiAPOA1, antiHCII, and antiCUBN identified as the most significant for these components (eigenvalues >0.7, Figure 4B). Additionally, when the multiple linear regression method was used to create the predictive model, the AUC values were 0.703, 0.820, and 0.828 for antiAPOA1 + antiНСII, antiAPOA1 + antiНСII, and antiAPOA1+ antiНСII + antiCUBN combinations, respectively. While antiCREBL3, antiORP150, and antiXPNPEP2 had less impact on the predictive value, including all six markers in the model only slightly increased the AUC values to 0.870 (Figure 6B). Thus, while multiple marker monitoring shows promise in discriminating between patients with moderate and severe COVID-19, three protein markers (antiAPOA1 + antiHCII + antiCUBN) suffice to achieve maximal diagnostic value. Furthermore, increasing the number of markers does not alter the sensitivity and specificity, with resulting AUC values falling within the same confidence interval. The accuracy of the model was confirmed by SVM, with values of approximately 0.833 for blood serum and 0.667 for urine. However, the lack of normalization of data to urine creatinine underscores the need to validate the model for this analyte.

In conclusion, the multiple regression model can be utilized in both research and clinical investigations of COVID-19 when multiple serum protein markers are employed. Nevertheless, our study found urine analysis to be inadequate due to the instability of protein marker levels in this biological material, particularly in a hospital environment where its composition can significantly influence results.

## 5 Limitations of the study

The sample size of enrolled patients is limited, which means that the observed and calculated parameters cannot be precisely extrapolated to the general population. These parameters might change when the sample size is increased. Additionally, the antibody level in the urine was not normalized by creatinine, so it might not accurately account for varying urine dilutions. These points should be considered as limitations.

## Data availability statement

The raw data supporting the conclusions of this article will be made available by the authors, without undue reservation.

## Ethics statement

The studies involving humans were approved by Independent Interdisciplinary Committee on Ethical Expertise of Clinical Trials (protocol 01-21, at 28.01.2021). The studies were conducted in accordance with the local legislation and institutional requirements. Written informed consent for participation in this study was provided by the participants' legal guardians/next of kin.

## Author contributions

YS: Writing – original draft, Validation, Conceptualization, Data curation, Formal analysis, Investigation, Software, Visualization. OY: Writing – original draft, Data curation, Supervision. AG: Investigation, Resources, Writing – original draft. IM: Writing – original draft, Supervision. NK: Methodology, Supervision, Writing – original draft. OL: Resources, Supervision, Writing – original draft. AZ: Methodology, Resources, Supervision, Writing – original draft. VE: Data curation, Project administration, Supervision, Writing – original draft. ASo: Data curation, Methodology, Supervision, Writing – original draft. PB: Data curation, Resources, Writing – original draft. DA: Data curation, Investigation, Resources, Writing – original draft. ASe: Data curation, Formal analysis, Investigation, Writing – original draft. SE: Investigation, Writing – original draft. AK: Data curation, Formal analysis, Investigation, Writing – original draft. PS: Data curation, Supervision, Writing – original draft. AF: Methodology, Resources, Writing – original draft. MD: Data curation, Resources, Writing – original draft. ST: Data curation, Project administration, Supervision, Writing – review & editing. JB: Conceptualization, Methodology, Project administration, Supervision, Writing – review & editing.
